# Modulation of the Gut Microbiota by Olive Oil Phenolic Compounds: Implications for Lipid Metabolism, Immune System, and Obesity

**DOI:** 10.3390/nu12082200

**Published:** 2020-07-23

**Authors:** Marta Farràs, Laura Martinez-Gili, Kevin Portune, Sara Arranz, Gary Frost, Mireia Tondo, Francisco Blanco-Vaca

**Affiliations:** 1 Institut de Recerca de l’Hospital Santa Creu i Sant Pau, Institut d’Investigacions Biomèdiques (IIB) Sant Pau, 08041 Barcelona, Spain; 2 CIBER de Diabetes y Enfermedades Metabólicas Asociadas (CIBERDEM), 08907 Barcelona, Spain; fblancova@santpau.cat; 3 Division of Systems Medicine, Department of Metabolism, Digestion and Reproduction, Faculty of Medicine, Imperial College London, London SW7 2AZ, UK; laura.martinez@imperial.ac.uk; 4 AZTI, Food Research, Basque Research and Technology Alliance (BRTA), Parque Tecnológico de Bizkaia, AstondoBidea, Edificio 609, 48160 Derio, Spain; kportune@azti.es (K.P.); sarranz@azti.es (S.A.); 5 Division of Digestive Diseases, Department of Metabolism, Digestion and Reproduction, Faculty of Medicine, Imperial College London, London SW7 2AZ, UK; g.frost@imperial.ac.uk; 6 Hospital de la Santa Creu i Sant Pau, Servei de Bioquímica-Institut d’Investigacions Biomèdiques (IIB) Sant Pau, 08041 Barcelona, Spain; mtondo@santpau.cat; 7 Departament de Bioquímica i Biologia Molecular, Universitat Autònoma de Barcelona, 08193 Barcelona, Spain

**Keywords:** gut microbiota, immune system, lipids, obesity, olive oil, phenolic compounds

## Abstract

There is extensive information of the beneficial effects of virgin olive oil (VOO), especially on cardiovascular diseases. Some VOO healthy properties have been attributed to their phenolic-compounds (PCs). The aim of this review is to present updated data on the effects of olive oil (OO) PCs on the gut microbiota, lipid metabolism, immune system, and obesity, as well as on the crosstalk among them. We summarize experiments and clinical trials which assessed the specific effects of the olive oil phenolic-compounds (OOPCs) without the synergy with OO-fats. Several studies have demonstrated that OOPC consumption increases *Bacteroidetes* and/or reduces the *Firmicutes*/*Bacteroidetes* ratio, which have both been related to atheroprotection. OOPCs also increase certain beneficial bacteria and gut-bacteria diversity which can be therapeutic for lipid-immune disorders and obesity. Furthermore, some of the mechanisms implicated in the crosstalk between OOPCs and these disorders include antimicrobial-activity, cholesterol microbial metabolism, and metabolites produced by bacteria. Specifically, OOPCs modulate short-chain fatty-acids produced by gut-microbiota, which can affect cholesterol metabolism and the immune system, and may play a role in weight gain through promoting satiety. Since data in humans are scarce, there is a necessity for more clinical trials designed to assess the specific role of the OOPCs in this crosstalk.

## 1. Introduction

The Mediterranean diet has been demonstrated to reduce the risk of cardiovascular diseases (CVD), cancer, neurodegenerative diseases, and diabetes [1]. Concretely, the PREDIMED study, the largest Mediterranean-diet trial, has extensively studied the effects of this diet on such diseases and the outcomes reviewed in the present work in cardiovascular risk individuals (Supplementary Table S1). The main source of fat in the Mediterranean diet is virgin olive oil (VOO) which also presents numerous beneficial effects, especially on cardiovascular diseases [2,3]. Improvement of blood pressure and protection of the intestinal mucosa have been attributed to olive oil (OO) fatty acids, especially to oleic acid, the most abundant lipid in OO [4,5]. Nevertheless, other healthy properties have also been attributed to the phenolic compounds (PCs) present in VOO. In that sense, several studies have reported beneficial effects of the olive oil phenolic-compounds (OOPCs). The Eurolive clinical trial with 200 healthy participants demonstrated that OOPCs increased high-density lipoprotein cholesterol (HDL-c) and decreased lipid oxidative damage in a dose-dependent way [6]. In this regard, in 2011, the European Food Safety Authority, recommended the consumption of OO with at least 5 mg of hydroxytyrosol (HT) (the main OOPC) and its derivatives per 20 g of OO to protect from lipid oxidative damage. Further, OOPCs enhance the expression of cholesterol-efflux related genes in circulating white blood cells of pre-/hypertensive patients [7] and improve endothelial function and HDL characteristics in hypercholesterolemic patients [8,9]. Altogether, these lipid profile improvements after VOO consumption could contribute to the cardioprotective properties observed in the Mediterranean diet.

Since it is well known that changes in the gut microbiome influence CVD [10], studying the effects of OOPCs on the gut microbiome may shed some light on additional involved mechanisms. To date, few studies have addressed this issue. It has been described that diet influences the intestinal microbiota, producing various enterotypes [11] with a predictable composition [12] according to the type of food ingested. Specifically, dietary fat intake has been described as one of the factors influencing gut microbiota composition [13], such as the *Firmicutes*/*Bacteroidetes* ratio, which is decreased in overweight and obese subjects compared to normal weight controls [14,15,16,17]. Protein, carbohydrates, and indigestible fiber are also relevant substrates and modulators of gut microbiota, as well as dietary PCs, which can be catabolized and transformed by gut microorganisms changing their biological activity and availability for the host. PCs enhance the growth of bacteria associated with healthy metabolic markers such as *Bifidobacteria*, *Lactobacillus*, *Akkermansia* spp., and *Faecalibacterium* spp. This prebiotic effect has been observed with higher intake of foods rich in PCs such as cocoa, pomegranate, nuts, grape, wine, tea, and berries [18].

Microorganism abundance and diversity across the human gastrointestinal tract increase longitudinally from the stomach to the colon [19,20], with more than 1000 different species of bacteria in the human intestinal tract and around 38 trillion bacteria resident in the colon [21]. Gut microbes have relevant roles in host metabolic and immune homeostasis, and a disturbed microbiota, altered intestinal permeability, and inflammation have been observed in obesity [22], insulin resistance [23], and CVD. Furthermore, obesity and CVD share some of the microbiota alterations found in these studies [24].

Gut microbial-derived metabolites may also play a key role in immune homeostasis and host susceptibility to immune-mediated pathologies and alterations [25].For example, short chain fatty acids (SCFA) produced from the diet are important modulators of the immune system and may play a role in weight loss in obesity through promoting satiety [26]. Another example is the microbial-host cometabolite trymethylamine-N-oxide (TMAO) which affects lipid metabolism and atherosclerosis [27].

Overall, OOPCs from the diet can influence the gut microbiota and its metabolism, thereby affecting human health and disease. The aim of this review is to present up-to-date information on the OOPC’s effects on gut microbiome, lipid metabolism, immune system, and obesity, as well as the crosstalk among them.

## 2. Olive Oil Composition

The Mediterranean diet is particularly rich in OO, fruits, vegetables, legumes, nuts, and seafood. In contrast, Western diets are characterized as being rich in processed meats, sugar-sweetened soft drinks, and refined grains [28]. OO is the main fat source in the Mediterranean diet which, as previously stated, is characterized for improving cardiovascular risk and cardiovascular and total mortality risk [2,3]. The major compounds of OO are fatty acids (98%), specifically, monounsaturated fatty acids (55–83%; oleic acid and palmitoleic acid), polyunsaturated fatty acids (11–42.5%; linolenic acid, linoleic acid), and saturated fatty acids (8–25.1%; palmitic acid, myristic acid, stearic acid). The minor compounds include lipophilic PCs (tocopherols and tocotrienols), hydrophilic PCs (phenolic acids, phenolic alcohols, secoirioids, lignans, and flavones), volatile compounds, pigments (chlorophylls), hydrocarbons (squalene, β-carotene, lycopene), sterols (β-sitosterol, campesterol, estigmasterol), triterpene and aliphatic alcohols, and nonglyceride esters (alcoholic and sterol compounds, waxes). These minor compounds help to preserve vitamin E and increase OO nutritional and sensorial properties. The principal minor compound of OO is HT, a PC with health properties. Another OO compound with extensively demonstrated health properties is oleuropein, which is found mainly in olive leaves but also in small quantities in OO itself [29].

VOO and extra VOO are obtained by direct pressing or centrifugation of the olives and it is rich in PCs (ranging from ~150 to 400 ppm in currently marketed commercial OOs). VOO also has a very good sensorial quality due to its low acidity index (<0.8%). A major beneficial effect of VOO is the reduction of LDL susceptibility to oxidation [30]. Refined OO, on the other hand, is obtained by mechanical extraction from unsuitable fruits and the majority of OOPCs in this type of OO are lost during refinement procedures [31]. In general, VOOs currently in the market do not achieve the quantity of PCs recommended by European Food Safety Authority. Therefore, functional enriched OOs are being manufactured to increase PC concentrations in VOOs without increasing its fat content. Moreover, functional OOs enriched with complementary PCs have more beneficial effects than OOs enriched with only OOPCs [32].

## 3. Olive Oil Phenolic Compounds and Gut Microbiota

### 3.1. Metabolism of OOPCs by Gut Microbiota

Effects of single OOPCs on gut microbiota have been described in the literature. The studies of this section are shown in Table 1.

Complex phenols undergo hydrolysis in the stomach due to its acidic environment, thereby increasing the proportion of simple phenols, such as tyrosol and HT, that will reach the duodenum [33]. While most of the simple phenols are rapidly absorbed in the small intestine, some simple and complex phenols reach the large intestine, where they can be metabolized by resident microbiota. Studies performed with batch fermentation of human colonic microbiota observed that oleuropein could be catabolized into HT [33], which in turn could be further oxidized into 2-(3′,4′-dihydroxyphenyl)acetic acid, followed by a dehydroxylation to 2-(4′-hydroxyphenyl)acetic acid and phenylacetic acid. Tyrosol could be oxidized into 2-(4′-hydroxyphenyl) acetic acid [34]. This latter study also detected HT acetate as another microbial metabolite product of oleuropein metabolism, which could either be hydrolyzed back into HT or converted to 3-(4′-hydroxyphenyl) propionic acid.

Isolated strains have been cultured to identify microbes responsible for such reactions. When grown in vitro, bacteria from the genera *Lactobacillus*, *Bifidobacteria*, and *Enterococcus* hydrolyse oleuropein to form HT [35]. Among the *Lactobacillus* genus, *Lactobacillus plantarum* is able to catabolize oleuropein into HT, as well as carry out the decarboxylation of protocatechuic acid into catechol [36] and hydroxycinnamic acids, like p-coumaric acid, into 4-ethylphenol and 4-ethylguaiacol [37,38].

The metabolism of less abundant OOPCs has also been assessed. Microbe suspensions from human faecal slurries are able to degrade apigenin, an OOPC, into different products such as 3-(4′-hydroxyphenyl) propionic acid [39]. The same study also showed excretion of apigenin microbial products in urine and faeces of germ-free rats humanized with human microbiota. The presence of PCs in serum, faeces, and urine has been measured after oral administration of apigenin, luteolin and other flavonoids by gavage into mice treated or not with antibiotics. After oral administration, serum flavonoid concentrations were not different between the two groups, indicating no differences in intestinal absorption. However, mice preserving their native microbiota excreted higher levels of phenolics in faeces and urine [40]. *Clostridium orbiscindens* [41] and a strain from the *Lachnospiraceae* family isolated from human faeces [42] are among identified microbes that are able to metabolize apigenin and luteolin.

Caffeic acid is another OOPC which is found in many other plants, mostly esterified with quinic acid in the form of chlorogenic acid. In human microbiota colonic cultures, caffeic acid can be catabolized to 3-hydroxyphenylpropionic and benzoic acids [43,44]. Ferulic acid can also be catabolized to 3-hydroxyphenylpropionic acid in colonic batch fermentations, sharing some of the catabolic pathways and products previously described [45].

Acteoside is a complex phenol made of a disaccharide, HT, and caffeic acid, which can be catabolized into its constituents HT and caffeic acid by human colonic cultures [46]. However, it is not yet known which microbial species are able to use acteoside as a substrate.

Pinoresinol, a lignan present in OO, is reduced to lariciresinol by *Enterococcus faecalis* [47] and *Eggerthellalenta* [48], two species commonly present in the gut microbiota.

Syringic acid can be demethylated to gallic acid by Peptostreptococcusproductus, Eubacteriumlimosum, Eubacteriumcallanderi, and Butyribacterium methylotrophicum [48].

The gut microbiome also has the potential to decarboxylate gallic acid into pyrogallol. Gallic acid decarboxylase gene was found in species belonging to the phyla *Firmicutes*, *Proteobacteria*, and *Actinobacteria* [49].

Little is known about the impact of OOPCs on microbiota located in the small intestine. In this regard, a couple of studies using microbes isolated from pig caeca observed that they were able to metabolize apigenin glycosides [50] and catabolize luteolin into 3-(3-hydroxyphenyl)-propionic acid [51]. To our knowledge, no studies have assessed OOPC microbial metabolism in the jejunum or ileum so far.

### 3.2. Antimicrobial Properties of OOPC

The studies of this section are shown in Table 1.

OO and its phenol extracts have long been known to have in vitro antimicrobial properties against common pathogens like *Staphylococcus aureus* and *Escherichia coli* [52,53] and also against in vitro cultured commensal bacteria, such as *Lactobacillus acidophilus* and *Bifidobacterium bifidum* [54].

Pure OOPCs have also been tested against isolated gut microbial strains or faecal slurries in vitro [55]. Caffeic acid inhibited the growth of both common pathogenic and commensal strains. In contrast, gallic acid did not have antimicrobial properties but rather could actually promote the growth of some pathogens like *Escherichia coli* O157:H7 [56]. Interestingly, microbial products from the metabolism of these two phenols also inhibited the growth of many microbial species.

Antimicrobial activity of many OOPCs was confirmed by Karaosmanoglu et al. using cultured bacterial strains. Cinnamic, ferulic, 4-hydroxybenzoic, vanillic and syringic acids as well as luteolin, vanillin and tyrosol exhibited antimicrobial properties against *Escherichia coli* O157:H7, *Listeria monocytogenes* and *Salmonella enteritidis* [57].

Apigenin was able to reduce the growth of *Bacteroides galacturonicus* and *Enterococcus caccae*in isolated cultures but promoted *Enterococcus* growth, SCFA production, and microbial diversity when added to human faecal colonic batch cultures [58]. This discrepancy between isolated cultures and colonic batch fermentation shows the complexity of microbial community dynamics. In addition, another study using a similar experimental setting with faecal cultures from three different donors and 200 µM of apigenin (instead of 370 µM used in the previously mentioned study), did not detect changes in microbial composition or SCFA production [59]. The different doses of apigenin, the use of frozen versus fresh faeces in the batch cultures, respectively, and the interindividual differences of microbial composition between donors could be the main reasons for these discrepant findings. Interestingly, an indirect effect of apigenin on gut microbiota composition, relying on host NOD-like receptor pyrin domain-containing protein 6 (NLRP6) signaling, was observed in mice [60]. This shows the potential of OOPCs to act as bioactive molecules further modulating host pathways and microbial composition.

While many of these studies were in vitro, it is important to note that in vitro experimental settings may not realistically reflect the intestinal lumen environment, the different compounds’ bioavailability, and the microbial community dynamics.

### 3.3. Microbiota Composition Modulation by OOPC

Human nutritional studies assessing the relationship between OOPCs and gut microbiota are scarce and often use whole OO dietary supplementation with a poor control of the overall diet composition between participants. This creates two strong confounding sources. First, the gut microbiota composition can be influenced by both the lipidic content in OO [61] and second by the differing consumption of phenol-containing foods between participants which can change phenolic bioavailability. Therefore, it is difficult to draw clear conclusions on the specific impact of OOPC on the microbiome in humans without considering such confounders. The VOO and HDL functionality (VOHF) study was a randomized, controlled, double-blind, crossover clinical trial [32] in which hypercholesterolemic participants ingested one of three VOOs (25 mL/day) for three weeks: raw, supplemented with OOPCs or supplemented with PCs from both OO and thyme. Importantly, lipidic composition was the same among the oils tested and dietary intake was monitored. In a subsample of participants, a subset of preselected bacterial groups was quantified using fluorescence *in situ* hybridization (FISH) and fluorescence-activated cell sorting (FACS) techniques showing modest changes in microbial composition of *Bifidobacterium* spp. and *Parascardovia denticolens*. Nevertheless, microbial changes could be underestimated due to the low number of participants and the limited coverage and resolutive power of the probes used to quantify microbial composition [62].

In a preclinical study, mice were fed a chow or high-fat diet enriched in extra VOO, refined OO, or butter for three months [63]. Mice fed an extra VOO diet had an increase in abundance of bacteria from the families *Sutterellaceae* and *Erysipelotrichaceae* and a decrease in *Christensenellaceae* and *Prevotellaceae* when compared to chow diets. At the genus level, mice fed extra VOO had a decreased abundance in *Desulfovibrio* with respect to refined OO and a decrease in *Anaerophaga* and *Fusicatenibacter* and an increase in *Parasutterella* and *Marinilabilia* compared to the chow diet. However, as mentioned previously, changes in microbial composition between chow and extra VOO diets could also be attributed to differences in their lipidic content.

The *Firmicutes*/*Bacteroidetes* ratio is a biomarker of altered colonic microbial composition [15,16,17]. A diet high in lipids can increase this ratio, and dietary supplementation with OOPCs such as HT in mice fed with high fat diet does not restore this ratio but increases the abundance of the genus *Lactobacillus* [16,64,65]. Murine in vivo studies also assessed the effect of single OOPCs on gut microbiota and the *Firmicutes*/*Bacteroidetes* ratio. Supplementing caffeic acid in drinking water ameliorated symptoms of dextran sulfate sodium (DSS)-induced colitis, reduced the *Firmicutes*/*Bacteroidetes* ratio and increased abundance of *Verrucomicrobia* phyla [66] (Table 1). Microbial changes of caffeic acid supplementation in control mice were not determined. In apolipoprotein E knockout mice (APOE^−/−^) fed a high-fat diet, oral ferulic acid administration reduced the *Firmicutes*/*Bacteroidetes* ratio. However, the effect of oral ferulic acid on APOE^−/−^ mice fed a control diet was not assessed [67] (Table 1). Mice exposed to airborne particulate matter and gavaged with HT, exhibited an increase in *Ruminococcaceae* and *Mycoplasmataceae* bacterial families. Again, the effect of HT on microbiota composition in control mice was not assessed or shown [68] (Table 1).

Similar results were reported in studies in which gut microbiota diversity was analyzed. Higher biodiversity of the gut microbiota was observed in hypertensive rats after administration of VOO, although the complete microbial populations in these groups were not fully characterized due to the limited molecular techniques applied [64].

Overall, the summarized studies show the potential of OOPCs to modulate microbial composition and the capacity of gut microbes to metabolize OOPCs. However, the value of OOPCs and their microbial derivatives as potential therapeutic agents for metabolic diseases needs to be further investigated in larger randomized trials devoid of confounding sources such as OO lipids and PCs derived from other foods.

## 4. Effects of Olive Oil Phenolic Compounds on Lipid Metabolism and Gut Microbiota

Over the last two decades, an association between metabolic disorders and dyslipidemia with gut microbiota has been observed [69]. Many studies have demonstrated a positive association between low-density lipoprotein cholesterol (LDL-c) and CVD riskand a negative association with high-density lipoprotein cholesterol (HDL-c) [70,71]. In a cohort of 155 European women, it was shown that 66 metagenomic gene clusters from gut microbiota were associated with serum triglyceride (TG) concentration, two with HDL-c concentration, and none with LDL or total cholesterol concentration [72]. One *Clostridiales* metagenomic gene cluster was negatively correlated with TG but positively correlated with HDL-c. A study with a large cohort of 893 healthy subjects described that gut microbiota explained 6.0% of the variation for TG, 4.0% for HDL-c, and 4.5% for body mass index (BMI), independently of age, sex, and genetics [73]. Individuals with high circulating TG and low HDL-c have lower microbial diversity and functional richness, high abundance of *Actinobacteria* phylum, and lower abundance of *Proteobacteria* and *Bacteroidetes* phyla. In addition, a study in obese adolescents reported that the genus *Ruminococcus* was associated with total cholesterol, while *Parvimonas* was negatively associated with LDL-c, and HDL-c was positively associated with *Mogibacteriaceae* [74].

Several studies have reported effects of OOPCs on cholesterol metabolism; nevertheless, works assessing OOPCs, microbiota, and lipids altogether are scarce. In a subsample from the VOHF study, no changes in faecal cholesterol concentrations were observed after different VOO interventions. Nevertheless, an increase in coprostanone was shown after the OOPC-enriched VOO intervention [4]. Coprostanone is one of the products generated when gut microbiota metabolize cholesterol. Although PC supplementation affects gut microbiota composition and reduces cholesterol in the blood [75], no changes in blood cholesterol were observed in the study by Peláez et al. [62]. Since cholesterol is the precursor of bile acids, it would be interesting to assess whether bile acid profiles changed across the different dietary interventions.

A number of studies have indicated that oxidized LDL is associated with the severity of acute coronary events [76] and can be considered a coronary heart disease biomarker [77]. Investigations have described that LDL-c concentration and oxidized LDL decreased linearly with the PC content of various OOs [6]. By contrast, in postprandial studies in healthy volunteers, oxidized LDL increased after OO intake with high concentrations of OOPCs [8] or after high doses of VOO [78]. In a subsample of the VOHF study, the intake of a functional OO enriched with OOPC and thyme PC decreased oxidized LDL and increased *Bifidobacteria* and *Parascardorvia* genera [62]. It has been previously described that *Bifidobacteria* abundance increases after consumption of other PC-containing foods (wine, wild blueberry, pomegranate peel, and cocoa) [79,80,81,82]. Furthermore, it has been observed that the serum lipid profile improved after *Bifidobacteria* and *Lactobacilli* mixed probiotic supplements were administered in clinical and preclinical studies [83,84]. The increase in *Bifidobacterium* could be responsible for the decrease in oxidized LDL. *Bifidobacterium* is a beneficial bacteria, a marker of gut microbiota balance, that helps maintain the intestinal barrier and uses oleuropein as a carbon source [17,23]. *Parascardovia* was also increased after the consumption of OO, indicating that these bacteria could also be related with the diminishing of oxidized LDL [62].

Other beneficial gut bacteria are lactobacillus. Species from *Lactobacillus* genus seem to be differentially affected by VOO. In hypertensive rats, the consumption of extra VOO consumption increased *Lactobacillus*. In this line, a study in high-fat diet-induced obese mice showed that the intake of HT (50 mg/kg) increased *Lactobacillus*, especially *L. johnsonii* [65]. In addition, it has beendescribed that probiotic bacteria can decrease total cholesterol and LDL-cholesterol [85,86]. In contrast, extra VOO consumption reduced some Lactobacillus species abundance, especially *L. animalis*, *L. taiwanensis*, and the genus *Lactococcus*, which correlated positively with weight [87]. In addition, olive-pomace-enriched biscuits decreased oxidized LDL and decreased the relative abundance of *Lactobacillus* [88]. Overall, contradictory results regarding the relationship with oxidized LDL and probiotic bacteria exist. Gut microbiota also influence the synthesis of cholesterol through regulating intestinal bile acid metabolism. *Bifidobacteria* among other microbes deconjugates bile acid salts [89], which are not absorbed and therefore excreted to faeces or further modified by bacteria. In order to compensate for the reduced bile acid availability, new bile acid synthesis occurs and consequently blood cholesterol levels decrease. In addition, it has been recently described that *Lactobacillus* could decrease blood cholesterol by diminishing fat absorption from intestine via the farnesoid X nuclear receptor [90]. However, no differences in either faecal bile acids or blood cholesterol were observed after OOPC intake in humans [62].

Prieto et al. [87] showed lower *Desulfovibrio* in the faeces of mice consuming extra VOO compared to an enriched butter diet. Nevertheless, in this study lower *Desulfovibrio* was not associated with lower total cholesterol levels. Total cholesterol levels correlated positively with *Anaerophaga*, *Parasutterella*, *Erysipelotrichaceae*, and *Olivibacter* and negatively with *Prevotella* and *Fusicatenibacter*. The HDL/LDL ratio negatively correlated with *Parasutterella* and *Fusicatenibacter*. In addition, increased levels of *Sutterellaceae*, *Marispirillum*, and *Mucilaginibacterdageonensis* were observed in mice consuming the extra VOO.

*Bacteroidetes* phylum is associated with atheroprotection and with reduction of intestinal permeability and plasmatic lipopolysaccharides (LPS). As previously stated, a number of in vivo studies demonstrated that extra VOO intake [87,91] and some OOPCs increased *Bacteroides* or decreased *Firmicutes/Bacteroides* ratio [66,67]. However, the ratio *Firmicutes*/*Bacteroidetes* did not change after HT consumption [16,64,65].

Metabolites produced by the gut microbiota can also exert effects on lipid metabolism. Trimethylamineoxide (TMAO) is a microbial-host cometabolite generated from dietary choline and L-carnitine, which are transformed to trimethylamine (TMA) by gut microbes, and then metabolized to TMAO by flavin monooxygenases in the liver. TMAO promotes atherosclerosis in animal models and is associated with CVD and adverse cardiac events in humans [92,93,94]. It has been described that TMAO inhibits the reverse cholesterol transport pathway. Inhibition of TMAO production is being explored as a potential therapeutic approach for atherosclerosis [95]. Nevertheless, no effects of OOPCs on TMAO are described in the literature. In contrast, OOPCs are able to affect other gut microbiota metabolites implicated on lipid metabolism, such as bioactive PC metabolites and SCFAs.

One of the bioactive PC gut microbiota metabolites that can affect lipid metabolism is protocatechuic acid. This metabolite enhances reverse cholesterol transport in mice [96] and inhibits LDL oxidation [62,97]. Protocatechuic acid was found elevated after the consumption of VOO enriched with OOPCs and thyme PCs, concomitant with a reduction of oxidized LDL [62]. The antioxidant activity of HT and other microbiota-derived compounds helps to avoid lipoprotein modifications that lead to atherosclerosis plaque formation [98].

A number of studies have shown that PC consumption induces changes in SCFA levels produced by gut microbiota [99], mainly acetate, propionate, and butyrate. Grapefruit PCs have been demonstrated to raise SCFAs in rats [100], while PCs of red wine and black tea reduced them [101]. These changes in SCFA generation could influence the cholesterol metabolism since acetate is the primary substrate for cholesterol synthesis [102], propionate is related to lipid metabolism [103], and butyrate reduces total cholesterol [64]. In a subsample of the VOHF study, ingestion of different VOOs did not affect the faecal quantities of propionic, acetic, and butyric acids [62]. Butyrate excretion in faeces of mice was increased after consumption of an OOPC extract-enriched diet in comparison with a control diet [104]. It has been reported that extra VOO consumption in hypertensive rats increases the cluster *Clostridium XIVa*, a strict anaerobic group. This bacterial group includes butyrate producers, which has been linked to reductions in total cholesterol as previously mentioned [64]. In this regard, extra VOO is also associated to *Clostridium cocleatum* [11,31]. On the contrary, a borderline decrease of *Clostridium cluster XI* was observed after an OOPC-enriched VOO diet versus a nonenriched VOO diet, and no changes were observed in total cholesterol after this intervention [62]. *Ruminococcus*, from the order *Clostridia*, was decreased in relative abundance after consumption of olive-pomace enriched biscuits in mildly hypercholesterolemic humans [88]. Thus, an increase of butyrate or gut bacteria that produce butyrate has been observed after OOPCs consumption in various studies. However, the majority of these studies did not analyze total cholesterol. Therefore, studies measuring both parameters are needed to obtain clear conclusions regarding the effects of butyrate on cholesterol metabolism. Furthermore, not all studies disentangle the possible effects exerted by OOPCs or OO lipids.

In conclusion, the beneficial effects of OOPCs on lipid metabolism could be partially explained by gut microbiota changes induced by OOPCs. OOPCs increase *Bacteroidetes* or reduce the *Firmicutes*/*Bacteroidetes* ratio, both associated with atheroprotection. Moreover, butyrate (which decreases cholesterol) or gut bacteria that produce butyrate increase after consumption of OOPCs. Finally, the increase of beneficial gut bacteria after consumption of OOPC such as *Bifidobacteria*, and in some cases *Lactobacillus*, could have a positive influence on lipid metabolism. Nevertheless, the relationship between oxidized LDL and *Lactobacillus* is heterogeneous across species, experimental setting, and host organism. Further clinical trials are needed to expand the knowledge on microbiota-OOPC-lipid homeostasis relationship.

In vivo studies which analyze the effect of OOPCs on microbiota and lipids at the same time are shown in Table 2.

## 5. Effects of Olive Oil Phenolic Compounds on Gut Microbiota and Immune System

The gut microbiome is extremely important in the development and maturation of a normal immune system. Diet can influence aspects of human biology by connecting nutrient metabolism, gut microbiota, and the immune system [105,106]. The gut microbiota contributes to the development of gut-associated lymphoid tissues (GALTs) which constitutes the most extensive and complex part of the immune system in the body), the polarization of gut-specific immune responses, and the prevention of colonization by pathogens. In turn, the composition of the microbiota is regulated by gut immune responses that are induced by commensal populations. Moreover, the alteration of the composition of the microbial composition and gut barrier function is observed in autoimmune and chronic inflammatory diseases [107,108]. As such, a lower *Firmicutes*/*Bacteroidetes* ratio in systemic lupus erythematosus patients has been described [109]. Conversely, Firmicutes are increased in rheumatoid arthritis [110] and Sjögren’s syndrome patients [111]. Overall, the crosstalk between GALT and the microbiota is a complex and tightly regulated process, which has been shown to be critical for mucosal tissue homeostasis, maintenance of mucosal barrier function, and protection against infectious and mucosal inflammatory diseases [112]. Furthermore, low bacterial species diversity has also been associated with increased incidence of inflammatory phenotype [113].

VOO exerts anti-inflammatory and immunomodulatory activities partially due to its content in PCs [62,114,115,116]. Extra VOO PCs are absorbed, metabolized, and distributed in the blood stream throughout the body, being potentially useful agents in modulating local and systemic inflammatory environments in immune-mediated inflammatory diseases (IMID) [117]. For this reason, the capability of PCs has been worthy of consideration as potential agents in the IMID management [34,117].

Works exploring the anti-inflammatory activity of OOPCs together with its role in the immune system and in the prevention and treatment of IMID have been recently reviewed [118]. The effects of extra VOO and its PCs positively influence the expression of pro- and anti-inflammatory mediators involved in IMID by modulating specific signaling molecules and related transcription factors. VOO ingestion has been shown to decrease the levels of inflammatory markers such as IL-6 [119], visfatin [120], TNF-α, IL-1β, COX-2 in plasma [121], IFN-γ in plasma and in the epididymal adipose tissue [122], TNF-α, IL-6, and IL-17 in splenocytes [123] and IL-1, IL-3, IL-8, and COX-2 in peripheral blood mononuclear cells [124,125]. The beneficial effects of extra VOO intake might also include improving the anti-inflammatory and antioxidant defenses by increasing the expression of cytokines such as IL-10 [121], the activity of the antioxidant GPX enzyme [126], and the antioxidant transcription factor Nrf2 [127]. Similar results have been observed with dietary supplementation of single OOPC components such as oleuropein and its derivative HT [128,129,130,131], tyrosol [132], and oleocanthal [133]. In vitro studies have demonstrated that most of the crossinteracting signaling pathways activated in IMID appear to be modulated by VOO PCs [124,134]. Nevertheless, existing data is not enough to issue specific nutritional guidelines for immune-mediated diseases. Appropriate clinical and epidemiological studies are necessary to further evaluate the contribution of OOPCs in IMID management.

It is also of our interest to review studies that explore the effects of OOPCs consumption on the immune system by means of microbiota. In that sense, in a subsample of the VOHF study, it was investigated whether PCs influenced mucosal and systemic immunity. Hypercholesterolemic patients who ingested OO enriched with PC for three weeks presented higher IgA-coating bacteria and C-reactive protein (CRP) [112]. Reasonable explanations for these findings were either a change in the composition and metabolic output of gut bacteria or qualitative changes in species that would stimulate the production and secretion of IgA. However, the concomitant increase in plasma CRP seems inconsistent with the antioxidant and anti-inflammatory effects associated with OOPCs. It has been previously stated that high doses of exogenous antioxidant compounds may be toxic [135]. It is possible that the unusually high amount of PCs included in the OO consumption sustained for three weeks could be responsible for the unexpected effect in CRP. CRP levels have also been shown to raise when circadian rhythm is altered [136], in type-2 diabetes [137], and with dietary habits or fast/fed status [138], factors that might have contributed to the observed increase in CRP. Another randomized controlled trial which tested an OOPC-enriched VOO in pre-/hypertensive individuals in a postprandial situation, demonstrated that this treatment improved proinflammatory biomarkers such as VCAM-1 and PAI-1. However, this study did not examine the gut microbiota [139].

Fish oil and VOO consumption has also been proven effective on decreasing joint pain intensity and ameliorating clinical and laboratory parameters of rheumatoid arthritis [140]. Several other studies related to psoriasis have demonstrated an inverse association of disease severity and CPR levels following extra VOO consumption [141]. Improvement of psoriasis signs also followed application of herbal preparation containing high percentage of OO [142].

Some of the observed discrepancies among the studies could be explained by the different PC concentrations in VOOs, by the different healthy background of the included participants, or by the design of the clinical trial (long-term vs. acute). Further studies should be designed in order to address the controversy regarding the proinflammatory biomarkers.

Probiotics have been considered as adjuvant therapy for autoimmune diseases. Potential mechanisms supporting this include increased mucus secretion, antimicrobial peptide production, enhancement of the gastrointestinal–epithelial barrier function, and improvement of gut microbiota–mucosal immune cell crosstalk [143]. OOPCs act as potential prebiotics for the growth of certain colonic bacterial strains such as *Lactobacillus* and *Bifidobacterium* [33,62] and increase gut bacteria diversity, which is associated with healthier phenotypes. OOPCs could be protective for IMID since probiotics are beneficial for immune system and low bacterial species diversity has been associated with an inflammatory phenotype [113].

Furthermore, metabolites generated by gut microbiota are able to influence the immune system. Reduced butyrate has been found in IMID such as irritable bowel disease [144]. As previously stated, a number of studies demonstrated that PCs can modulate gut microbiota composition as well as their SCFA production. Concretely, increased butyrate or gut bacteria which produces butyrate has been observed after PCs and OOPCs consumption in various studies [64,104]. Moreover, irritable bowel disease patients have increased *Fusobacterium*, *Escherichia*, and *Proteobacteria* and decreased *Bacteroides*, *Bifidobacterium*, and *Clostridium* groups *IV* and *XIVA*. Several meta-analysis and cohort studies have demonstrated that fecal microbiota transplantation improves the bowel movement in affected patients and colitis animal models [145,146,147,148]. OOPC could also be beneficial in these patients since they increase *Bacteroidetes* and *Bifidobacterium* [62,66,67].

In conclusion, some immune disorders are characterized by low bacterial species diversity and low levels of butyrate. OOPCs could be a potential nutritional tool to improve the inflammatory status since they increase gut-bacteria diversity, butyrate concentrations, and beneficial microbes. Clinical trials in IMID patients are needed to confirm these hypotheses.

In vivo studies which analyze the effect of OOPCs on microbiota and the immune system at the same time are shown in Table 2.

## 6. Effects of Olive Oil Phenolic Compounds on Gut Microbiota and Obesity and Associated Morbidities

Obesity is a complex, multifaceted medical condition in which body fat accumulates in adipose tissue and throughout the body, with individuals exceeding a BMI of >30 kg/m2 being considered as obese. This chronic condition is often associated with several harmful comorbidities, such as Type 2 diabetes, nonalcoholic fatty liver disease, cardiovascular and neurodegenerative diseases, and several types of cancer [149,150]. The intestinal microbiota has been identified to be a potentially important player in the development, exacerbation, and/or alleviation of obesity and related comorbidities. Dietary PCs have been associated with palliating symptoms of obesity and related conditions such as metabolic syndrome. Multiple studies have shown a negative association between PC consumption and weight gain [151]. PCs from green tea, mainly epigallocatechin gallate, caffeoylquinic acid, grape seed proanthocyanidins, resveratrol from grapes or wine, anthocyanins and other flavonoids have also been inversely associated with both BMI and other clinical markers of metabolic syndrome [152]. Several studies found that weight gain, waist circumference, and blood pressure decreased after extra VOO consumption, in contrast with other vegetable oils [153]. Oleuropein, a phenolic acid found in VOO, has been characterized as having antidiabetic, antiatherosclerotic, and anti-inflammatory properties in humans [6]. HT, the main OOPC, downregulated the expression of PPAR-α and PPAR-γ, leading to a reduction in adipocyte size in in vitro studies [154,155]. HT could modulate inflamed human adipocyte gene expression by regulating the microRNAs miR-155-5p, miR-34a-5p, and let-7c-5p expression, leading to a reduction of oxidative stress and NF-kβ inhibition [156], which is a key pathway involved in systemic inflammation in obesity. HT also improves inflammation, insulin resistance, and hepatic steatosis by reducing endoplasmic reticulum stress and by regulating the JNK/IRS pathway in high-fat diet-induced obese mice [157]. Other mechanisms have been proposed for the antiobesogenic effects of PCs, including reducing nutrient intake by the gastrointestinal tract through digestive enzyme inhibition, reduced adipogenesis, alteration of glucose homeostasis, promotion of fat oxidation, and increasing energy expenditure via increased thermogenesis (reviewed in [151,158]). The gut microbiota has been shown to participate in increasing energy extraction and fat deposition in the host [159], as well as modulate host metabolism, inflammatory status, gut barrier integrity, and satiety [160]. Efforts to identify a specific gut microbiota taxonomic signature associated with the development of obesity have revealed large interindividual differences [161], although a core microbiome, or enterotype, has been associated with lean phenotypes. Deviations to this core, a dysbiosis, may be found in conditions such as obesity [162]. Initial studies on the composition of the gut microbiota in the obese phenotype indicated an increased ratio of the bacterial phyla *Firmicutes*/*Bacteroidetes* [14,163], although other studies have either not observed this trend or even observed opposite trends [72,164,165]. Effects of OOPCs on the *Firmicutes*/*Bacteroidetes* ratio are limited, as only a few studies have shown that administration of purified caffeic acid and ferulic acid, which are minor components of PC in extra VOO, reduce this ratio in mice [66,67]. However, other major PCs present in VOO, such as HT, showed no differences in this ratio [65]. Reduced bacterial species diversity and richness in the gut microbiota have also been associated with obesity [162,166,167], although there is also some evidence of increased diversity in at least some obese patients [168]. Low bacterial species diversity has also been associated with increased incidence of adiposity and insulin resistance [169]. Positive effects of OOPC administration on increasing microbial diversity have been observed in rodents, as previously stated. In high-fat diet-induced obese mice, the supplementation of HT restored microbial diversity back to comparable levels with the control group fed a standard chow diet [65]. In a recent study, mice fed a high-fat diet supplemented with either extra VOO or flaxseed oil, displayed significantly higher microbial diversity, as compared to mice fed a high-fat diet without these supplements [170]. However, it is unclear in this study whether these beneficial effects were directly attributable to the lipids or PCs present in the VOO.

OOPCs can also serve as prebiotics for the growth of certain beneficial colonic bacteria such as *Lactobacillus* and *Bifidobacterium* [33], both of which are bacterial genera with potential antiobesity effects [171,172]. Furthermore, several OOPCs have been shown to stimulate the growth of SCFA-producing bacteria as well as increase the production of SCFAs, as previously indicated. SCFAs are produced by gut microbiota fermentation of specific dietary sources, such as different types of indigestible fiber, which have recently been implicated in influencing host appetite and food intake [173]. SCFAs (primarily butyrate) interact with enteroendocrine cells via communication between the so called gut-brain axis to produce paracrine and/or endocrine signaling via glucagon-like peptide-1, cholecystokinin, peptide YY3-36, pancreatic polypeptide, and oxyntomodulin [173,174]. Furthermore, propionate, another important SCFA produced by gut microbiota in the colon, increases intestinal gluconeogenesis which increases satiety and improves glucose homeostasis [175]. PCs, including HT, resveratrol and anthocyanins are also known to stimulate the enzyme 5′ AMP-activated protein kinase, which is involved in regulating cellular energy homeostasis [158], possibly via an interaction with the gut microbiota to promote production of SCFAs [176]. Additionally, an increase in the enzyme 5′ AMP-activated protein kinase and hormone-sensitive lipase and phosphorylated lipase was observed in adipocytes exposed to HT [68]. It is important to note that although there is a large amount of evidence of beneficial effects of butyrate on alleviating diet-induced obesity and insulin resistance, human observational studies have also shown higher amounts of fecal excretion of SCFAs in obese subjects [164,177,178] and increased excreted SCFAs with consumption of high calorie diets [179]. SCFAs could therefore also contribute to adiposity by providing a substantial amount (10%) of the daily energy requirement in humans [180]. These factors complicate the role of SCFAs in diet-induced obesity and thus warrant more studies.

Obesity is often characterized by low grade intestinal inflammation caused by increased permeability of the intestinal epithelium, which can lead to the passage of microbially-derived compounds such as LPS into circulation, thereby causing metabolic endotoxaemia, and inflammatory disorders [181]. Related with intestinal inflammation, hydroxytyrosyl acetate (HT-Ac), also present in VOO, has been reported to exhibit gut anti-inflammatory effects [33] in DSS-induced acute colitis in mice [182].

Both the gut microbiota and PCs have been demonstrated to play roles in modulating the intestinal barrier. For instance, changes in intestinal gut microbiota caused by high-fat Western diets cause decreased production of SCFAs, host antimicrobial peptides, mucus production, and tight-junction production, which all lead to the increased intestinal permeability observed in obesity [181,183]. OOPCs contribute to maintaining gut barrier integrity by upregulating the expression of genes involved in maintaining tight junctions between intestinal cells, modulating the oxidative status of the intestinal epithelial layer, as well as the inflammatory and immune response [23,170]. Administration of HT to high-fat diet-induced obese mice showed increased gene expression for the tight junction associated proteins ZO-1 and occludin, as well as reduced levels of plasma LPS and inflammatory cytokines in the liver, all supporting a role for HT in promoting intestinal barrier integrity [65].

*Akkermansia muciniphila*, a mucin-degrading bacterium that is highly prevalent in healthy humans in the mucus layer covering the intestinal epithelium, is well-known for its inverse association with obesity, diabetes, and inflammation [151]. Furthermore, *A. muciniphila* treatment in mice reverses high-fat diet-induced metabolic endotoxemia, adipose tissue inflammation, and insulin resistance, restoring mucus thickness [184]. Pasteurized *A. muciniphila* treatment in humans improved insulin sensitivity, reduced plasma total cholesterol and blood markers of liver dysfunction and inflammation, and slightly decreased body weight [185]. In ulcerative colitis mouse models, administration of caffeic acid, present in VOO, was shown to reduce colonic infiltration of T-cells, neutrophils and proinflammatory cytokines, restoring the species richness and drastically increasing the proportion of *Akkermansia* [66]. Increased growth of *A. muciniphila* has also been associated with the consumption of prebiotic fructo-oligosaccharides, as well as certain dietary PCs found in black tea, red wine grape extracts, cranberry extracts, and other berries [151,186]. Although the exact mechanism behind this increase in *A. muciniphila* from PCs is unclear, it could be a result of an increase in the production of mucus is observed after PC administration, which could provide a major energy source for the bacterium [187]. Furthermore, the antioxidant properties of PCs could further propagate the anaerobic conditions required for successful growth of this bacterium [188].

In conclusion, evidence from in vitro and preclinical studies has demonstrated that OOPCs could be a potential nutritional tool to modulate the gut microbiome by increasing microorganism diversity and beneficial bacteria in obesity. However, nutritional clinical trials specifically designed to avoid confounder dietary factors in obese patients are needed to confirm these hypotheses. Moreover, many microbial metabolites such as SCFAs remain unidentified and their roles in obesity remain to be elucidated. Future investigations to address a causal relationship and the underlying mechanism in the gut microbiome and diet interaction with a particular emphasis on the development of microbiome-targeted therapies for obesity prevention and treatment are needed.

In vivo studies which analyze the effect of OOPCs on microbiota and obesity are shown in Table 2.

## 7. Conclusions

**Scheme 1:**
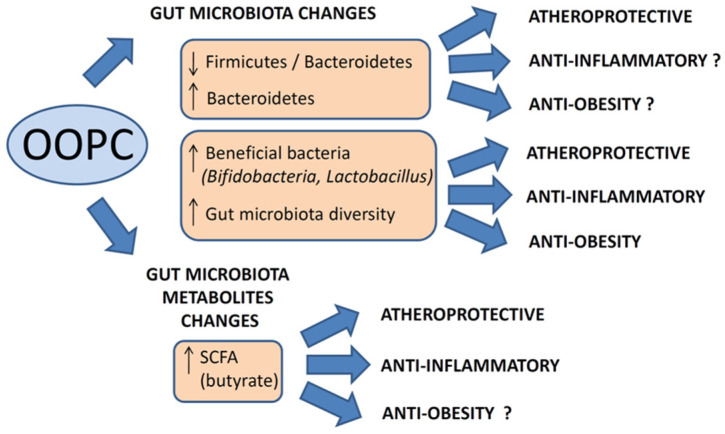
Effects of OOPC on gut microbiota and the implications for lipid metabolism, immune system, and obesity.

Overall, strong evidence suggesting a link between OOPCs, the gut microbiota, and their derived metabolites exists. Although many studies have been performed in vitro using faecal slurries or cultured bacteria, evidence suggests that OOPCs modulate microbial composition and metabolism and increase the diversity of the gut bacterial communities producing beneficial effects. Specifically, the consumption of OOPCs produces an increase of *Bacteroidetes* or a reduction of *Firmicutes*/*Bacteroidetes*, which is associated with atheroprotection. The observed increase in beneficial bacteria such as *Bifidobacteria*, and in some cases *Lactobacillus*, after OOPC administration could also have positive effects on atherosclerosis, immune disorders, and obesity. OOPC antimicrobial activity, bacterial cholesterol metabolism, and the different metabolites produced by bacteria are some of the factors and mechanisms implicated in these effects. Specifically, the consumption of OOPCs changes the SCFAs’ production by gut bacteria. SCFAs are involved in cholesterol metabolism, immune system regulation, and may play a role in weight gain in obesity through promoting satiety (Scheme 1). Despite the strong experimental evidence already accumulated, data from large-scale, long-term randomized clinical trials is still scarce. Furthermore, before conducting these clinical trials, it could be of interest to perform experiments with fecal microbiota transplantation in animal models in order to acquire relevant information regarding the restore of gut microbiota in each specific disorder. If successful, clinical trials designed to assess the specific role of the OOPCs in the above mentioned areas may provide the information needed for the development of new nutritional guidelines. These clinical trials should also clarify if it would be better to administer OOPCs alone with capsules or with a VOO matrix.

## Figures and Tables

**Table 1:** Studies which analyzed the effects of singleolive oil phenolic-compounds (OOPC) on gut microbiota and pathogens.

Compound	Experiment Setting	Effect	Reference
Apigenin	Caecum microbiota from pig (in vitro)	Degradation of apigenin monoglycoside into 3,4-dihydroxyphenylacetic acid or 4-hydroxyphenylacetic acid	[50]
	(1) Human faecal slurries (in vitro) or (2) Drinking water supplement in germ-free rats inoculated with human microbiota (in vivo)	(1) Conversion of apigenin into 3-(3,4-dihydroxyphenyl)propionic acid and 3-(3-hydroxyphenyl)propionic acid (2) Excretion of naringenin, phloretin, eriodictyol, 3-(3,4-dihydroxyphenyl)propionic acid, 3-(4-hydroxyphenyl)propionic acid, 4-hydroxycinnamic acid, and 3-(3-hydroxyphenyl)propionic acid in urine and faeces.	[39]
	*Lachnospiraceae* isolated strain from human faeces	Conversion of apigenin to 3-(4-hydroxyphenyl)propionic acid	[42]
	Bacteria strains or colonic communities (in vitro)	Different effects in bacterial growth and production of short chain fatty acids (SCFA)	[58]
	*Clostridium orbiscindens* isolated strain from human faeces	Conversion of apigenin to 3-(4-hydroxyphenyl)propionic acid	[41]
	(1) Mouse faecal suspensions (in vitro) and (2) mice gavaged with polyphenols with or without antibiotic treatment (in vivo)	(1) p-Hydroxybenzoic acid, p-Hydroxyphenylacetic acid, caffeic acid, 3-(4-Hydroxyphenyl)propionic acid and p-Coumaric acid were detected in the incubated faecal suspensions. (2) Serum protocatechuic and p-hydroxyphenylacetic acids were higher in the nontreated animals. Faecal and urine excretion were comparable and higher for nontreated mice. Vanillic acid was found in urine.	[40]
Benzoic/4-hydroxybenzoic acid	Bacterial strains in culture (in vitro)	Antimicrobial properties against *Escherichia coli* O157:H7, *Listeria monocytogenes* and *Salmonella enteritidis*	[57]
Caffeic acid	Human faecal slurries (in vitro)	Caffeic acid (free or esterified with quinic or tartaric acids) was converted to 3-hydroxyphenyl propionic acid and benzoic acid	[44]
	Bacterial strains in culture (in vitro)	Each strain was affected differently. Marked growth inhibition of pathogens, but different effects on other species.	[56]
	Mice with DSS-induced colitis supplemented caffeine in drinking water	Reduced the *Firmicutes*/*Bacteroidetes* ratio and increased abundance of *Verrucomicrobia* phyla	[66]
Cinnamic acid	Bacterial strains in culture (in vitro)	Antimicrobial properties against *Escherichia coli* O157:H7, *Listeria monocytogenes* and *Salmonella enteritidis*	[57]
Coumaric acid	*Lactobacillus plantarum* cultured strains (in vitro)	Decarboxylation of p-coumaric acid into 4-ethylphenol and 4-ethylguaiacol	[37]
Demethyloleuropein	no results in Pubmed	not investigated in the context of microbiome	-
Ferulic	APOE^−/−^ mice gavaged with ferulic acid (in vivo)	Reduction of the *Firmicutes*/*Bacteroidetes* ratio	[67]
	Human faecal slurries (in vitro)	Conversion of ferulic acid into (hydroxy)phenylacetic and (hydroxy)benzoic acid derivatives	[45]
	Bacterial strains in culture (in vitro)	Antimicrobial properties against *Escherichia coli* O157:H7, *Listeria monocytogenes* and *Salmonella enteritidis*	[57]
Gallic acid	Bacteria strains or colonic communities (in vitro)	Conversion of gallic acid to pyrogallol by species pertaining to the phyla *Firmicutes*, *Proteobacteria* and *Actinobacteria*.	[49]
	Bacterial strains in culture (in vitro)	Each strain was affected differently. Marked growth inhibition of some pathogens like *Clostridioides difficile*, but promoted growth of others like *Escherichia coli* 0157:H7 or *Salmonella enterica Typhimurium*.	[56]
Hydroxytyrosol	Mice gavaged with hydroxytyrosol and exposed to particulate matter (in vivo)	Increase in *Ruminococcaceae* and *Mycoplasmataceae* families abundance	[68]
	Human faecal slurries (in vitro)	Hydroxytyrosol is oxidised into 2-(3′,4′-dihydroxyphenyl)acetic acid	[34]
Ligstroside	no results in Pubmed	not investigated in the context of microbiome	-
Luteolin	(1) Mouse faecal suspensions and (2) mice gavaged with polyphenols with or without antibiotic treatment	(1) p-Hydroxybenzoic acid, Protocatechuic acid, p-Hydroxyphenylacetic acid and 3-(4-Hydroxyphenyl)propionic acid were detected in the incubated faecal suspensions. (2) Serum protocatechuic and hydrocaffeic acids were higher in the nontreated animals. Faecal and urine excretion were comparable and higher for nontreated mice.	[40]
	Bacterial strains in culture (in vitro)	Antimicrobial properties against *Escherichia coli* O157:H7, *Listeria monocytogenes* and *Salmonella enteritidis*	[57]
	Pig caecum microbiota (in vitro)	Luteolin converstion into 3-(3-hydroxyphenyl)propionic acid	[51]
	*Lachnospiraceae* isolated strain from human faeces	Luteolin converstion into 3-(3,4-dihydroxyphenyl)propionic acid	[42]
Mixture: acteoside	Human faecal slurries (in vitro)	Metabolised into hydroxytyrosol and caffeic acid	[46]
Mixture: olive oil supplement	Olive oil dietary supplementation in humans (in vivo)	Changes in microbial composition of *Bifidobacterium* spp. and *Parascardoviadenticolens*	[62]
	Bacterial cultures treated with olive oils (in vitro)	Inhibition of growth of pathogens like *Escherichia coli* or *Clostridium perfringens* as well as commensals like *Lactobacillus acidophilus* and *Bifidobacterium bifidum*	[54]
Nuezhenide	no results in Pubmed	not investigated in the context of microbiome	-
Oleuropein	Human faecal slurries (in vitro)	Conversion of oleuropein into hydroxytyrosol	[33]
	Bacterial strains in culture (in vitro)	*Lactobacillus*, *Bifidobacteria* and *Enterococcus* genera hydrolyse oleuropein into hydroxytyrosol	[35]
Pinoresinol	*Eggerthellalenta* isolated culture (in vitro)	Conversion of pinoresinol to lariciresinol and secoisolariciresinol	[48]
	Human faecal slurries and *Enterococcus faecalis* strain (in vitro)	Conversion of pinoresinol to lariciresinol and secoisolariciresinol	[47]
Protocatechuic acid	*Lactobacillus plantarum* cultured strains (in vitro)	Decarboxylation of protocatechuic acid into catechol	[36]
Syringic acid	Bacterial strains in culture (in vitro)	Antimicrobial properties against *Escherichia coli* O157:H7, *Listeria monocytogenes* and *Salmonella enteritidis*	[57]
	Bacterial strains in culture (in vitro)	Syringic acid can be demethylated to gallic acid by *Peptostreptococcusproductus*, *Eubacteriumlimosum*, *Eubacteriumcallanderi* and *Butyribacteriummethylotrophicum*	[48]
Tyrosol	Human faecal slurries (in vitro)	Tyrosol is oxidised into 2-(4′-hydroxyphenyl)acetic acid	[34]
	Bacterial strains in culture (in vitro)	Antimicrobial properties against *Escherichia coli* O157:H7, *Listeria monocytogenes* and *Salmonella enteritidis*	[57]
(homo)Vanillic acid	Bacterial strains in culture (in vitro)	Antimicrobial properties against *Escherichia coli* O157:H7, *Listeria monocytogenes* and *Salmonella enteritidis*	[57]

**Table 2:** In vivo studies assessing the effects of OOPC on gut microbiota, lipid metabolism, immune system, and obesity and associated morbidities.

**OOPC on Lipid Metabolism and Gut Microbiota**					
**Treatments**	**Duration**	**Animal Model or Patients’ Health Status**	***N***	**OOPC Effects**	**Reference**
-Olive-pomace enriched biscuits (90 g) (1) -Isoenergetic control (2)	8 weeks Randomized Controlled Parallel Double-blind	Hypercholesterolemic patients	62 (1 = 34; 2 = 28)	Olive-pomace enriched biscuits: ↓oxidized LDL ↓*Lactobacillus* ↓*Ruminococcus* = gut microbiota diversity	[88]
-Extra VOO (1) -Enriched butter diet (2) -Standard diet (3)	12 weeks	Mice	26 (1 = 9; 2 = 9; 3 = 8)	Extra VOO: ↓*Desulfovibrio* ↑*Sutterellaceae* ↑*Marispirillum* ↑*Mucilaginibacter dageonensis* = lipids	[87]
-VOO enriched with OOPC and thyme PC (500 mg/kg PC) -VOO enriched with OOPC (500 mg/kg PC) -VOO	3 weeks (25 mL/day) Randomized Controlled Crossover Double-blind	Hypercholesterolemic patients	12	VOO enriched with OOPC and thyme PC: ↑*Bifidobacteria* ↑*Parascardovia* ↑protocateuchuic acid ↓oxidized LDL VOO enriched with OOPC: ↓*Clostridium cluster XI* (borderline) ↑coprostanone = total cholesterol	[62]
**OOPC on Immune System and Gut Microbiota**					
**Treatment**	**Duration**	**Animal Model OR Patients’ Health Status**	***N***	**OOPC Effects**	**Reference**
-VOO enriched with OOPC and thyme PC (500 mg/kg phenolics) -VOO enriched with OOPC (500 mg/kg phenolics) -VOO	3 weeks (25 mL/day) Randomized Controlled Crossover Double-blind	Hypercholesterolemic patients	10	VOO enriched with OOPC: ↑ mucosal immunity ↑IgA-coated bacteria	[62]
**OOPC on Obesity and Associated Morbidities, and Gut Microbiota**					
**Treatment**	**Duration**	**Animal Model or Patients’ Health Status**	***N***	**OOPC Effects**	**Reference**
-Normal fat diet (control) -Ferulic acid (30 mg/kg/day) -Simvastatin (5 mg/kg/day) -Normal saline	12 weeks	ApoE^−/−^ mice fed high-fat diets (male)	20 (5/group)	Ferulic acid: ↑serum TC, TG, and LDL-C ↑ α diversity ↓*Firmicutes*/*Bacteroidetes*	[67]
-High-fat extra-VOO -High-fat flaxseed oil -Low-fat -Lard	10 weeks	C57BL/6 J mice	20 (5/group)	High-fat extra-VOO: ↑ gut microbiota diversity ↓*Firmicutes* ↑ FoxP3 and IL-10 ↑ antimicrobial peptide RegIII	[170]
-PBS -Fine particular matter -Fine particular matter suspended in PBS (1 mg/mL) + HT (50 mg/kg/day)	4 weeks	C57BL/6 J mice (female)	45 (15/group)	Fine particular matter + HT: ↓visceral adipogenesis ↓oxidative stress ↓hepatic inflammation ↓NF-κβ activation ↓insulin resistance ↑ gut microbiota richness ↓pathogenic bacteria	[68]
-HT (+high fat diet) -HT fecal microbiota transplantation (+high fat diet) -High fat diet -Control (chow diet)	8 weeks	C57BL/6 J mice (male)	28 (7/group)	HT and HT fecal microbiota transplantation: ↓altered intestinal microbiota ↑ intestinal barrier function ↓endotoxin release ↓TNF-α, IL-1β, and IL-6 ↓lipid accumulation in liver = *Firmicutes*/*Bacteroidetes* ↓*Proteobacteria* ↓*Deferribacteres*	[65]
-Control -Water containing 2.5% DSS -Water containing 2.5% DSS + Caffeic acid (1 mM)	-DSS: 8 days -Caffeic acid: 15 days	Mice (control, DSS, DSS+caffeic acid)	30 (10/group)	Caffeic acid (in DSS colitis mice): ↓secretion of IL-6 ↓TNFα, ↓IFNγ ↓colonic infiltration of CD3^+^ T cells, CD177^+^ neutrophils, F4/80^+^ macrophages ↑ species richness ↑*Akkermansia* ↓*Firmicutes*/*Bacteroidetes*	[66]

CD, cluster of differentiation; FoxP3, forkhead box P3; HT, hydroxytyrosol; IFN, interferon; IL, interleukin; LDL-c, low-density lipoprotein cholesterol; NF, Necrosis factor; OOPC, olive oil phenolic compounds; TC, total cholesterol; TG, triglycerides; VOO, virgin olive oil; ↓, decrease; ↑, increase; =, no changes.

## Supplementary Materials

The following are available online at https://www.mdpi.com/2072-6643/12/8/2200/s1, Table S1: Results from PREDIMED study on lipid metabolism, immune system, and obesity.
