# Supplementary material for: Modulation of the Gut Microbiota by Olive Oil Phenolic Compounds: Implications for Lipid Metabolism, Immune System, and Obesity
Source: Nutrients. 2020 Jul 23;12(8):2200. doi: 10.3390/nu12082200 (PMC7468985; doi:10.3390/nu12082200)
Supplement: Supplementary file 1 [file nutrients-12-02200-s001.pdf]

Table S1. Results from PREDIMED study on lipid metabolism, immune system, and obesity.

DURATION	N	EFFECTS OF MEDITERRANEAN DIET SUPPLEMENTED WITH VOO	REFERENCE
5 years	7447	No changes in bodyweight<br>↓weight gain in central adiposity	[189]
1 year	1139	Participants in the highest tertile of changes in urinary phenolics:<br>↓VCAM-1<br>↓intercellular adhesion molecule<br>↓interleukin 6<br>↓tumour necrosis factor alpha monocyte<br>↓chemotactic protein 1	[190]
1 year	930	↓oxidized LDL	[3]
1-year	296	↑HDL functionality	[191]
1 year	210	↑ LDL resistance against oxidation<br>↓ LDL oxidative modifications<br>↑ LDL particle size<br>↑ LDL-cholesterol rich particles<br>↓LDL particles cytotoxicity	[192]
3 months	772	↑ HDL<br>↑ apo-A1<br>↓cholesterol/HDL<br>↓ LDL/HDL	[193]
3 months	551	↑apo-A1	[194]
3 months	106	↓C-reactive protein<br>↓monocytary adhesion molecules<br>↓chemokines	[193]
3-months	50	↑ VLDL-cholesterol<br>↑ VLDL-TG content<br>↑ TG/apoB ratio in VLDL	[195]

apo, apolipoprotein; TG, triglycerides;VCAM-1, vascular cell adhesion molecule 1; VOO, virgin olive oil;

189 Estruch, R.; Martinez-Gonzalez, M.A.; Corella, D.; Salas-Salvado, J.; Fito, M.; Chiva-Blanch, G.; Fiol, M.; Gomez-Gracia, E.; Aros, F.; Lapetra, J.; et al. Effect of a high-fat Mediterranean diet on bodyweight and waist circumference: A prespecified secondary outcomes analysis of the PREDIMED randomised controlled trial. *Lancet DiabetesEndocrinol.* **2019**, *7*, e6–e17, doi:10.1016/S2213-8587(19)30074-9.

190 Medina-Remon, A.; Casas, R.; Tressserra-Rimbau, A.; Ros, E.; Martinez-Gonzalez, M.A.; Fito, M.; Corella, D.; Salas-Salvado, J.; Lamuela-Raventos, R.M.; Estruch, R.; et al. Polyphenol intake from a Mediterranean diet decreases inflammatory biomarkers related to atherosclerosis: A substudy of the PREDIMED trial. *Br. J.Clin. Pharmacol.* **2017**, *83*, 114–128, doi:10.1111/bcp.12986.

191 Hernaez, A.; Castaner, O.; Elosua, R.; Pinto, X.; Estruch, R.; Salas-Salvado, J.; Corella, D.; Aros, F.; Serra-Majem, L.; Fiol, M.; et al. Mediterranean Diet Improves High-Density Lipoprotein Function in High-Cardiovascular-Risk Individuals: A Randomized Controlled Trial. *Circulation* **2017**, *135*, 633–643, doi:10.1161/CIRCULATIONAHA.116.023712.

192 Hernaez, A.; Castaner, O.; Goday, A.; Ros, E.; Pinto, X.; Estruch, R.; Salas-Salvado, J.; Corella, D.; Aros, F.; Serra-Majem, L.; et al. The Mediterranean Diet decreases LDL atherogenicity in high cardiovascular risk individuals: A randomized controlled trial. *Mol. Nutr. Food Res.* **2017**, *61*, doi:10.1002/mnfr.201601015.

193 Estruch, R. Anti-inflammatory effects of the Mediterranean diet: The experience of the PREDIMED study.*Proc. Nutr. Soc.* **2010**, *69*, 333–340, doi:10.1017/S0029665110001539.

194 Sola, R.; Fito, M.; Estruch, R.; Salas-Salvado, J.; Corella, D.; de La Torre, R.; Munoz, M.A.; Lopez-SabaterMdel, C.; Martinez-Gonzalez, M.A.; Aros, F.; et al. Effect of a traditional Mediterranean diet on apolipoproteins B, A-I, and their ratio: A randomized, controlled trial. *Atherosclerosis* **2011**, *218*, 174–180, doi:10.1016/j.atherosclerosis.2011.04.026.

195 Perona, J.S.; Covas, M.I.; Fito, M.; Cabello-Moruno, R.; Aros, F.; Corella, D.; Ros, E.; Garcia, M.; Estruch, R.; Martinez-Gonzalez, M.A.; et al. Reduction in systemic and VLDL triacylglycerol concentration after a 3-month Mediterranean-style diet in high-cardiovascular-risk subjects.*J. Nutr. Biochem.* **2010**, *21*, 892–898, 10.1016/j.jnutbio.2009.07.005.
